# Development of a Family-Based Mental Health Program for Runaway Adolescents Using an Intervention Mapping Protocol

**DOI:** 10.3390/ijerph17217794

**Published:** 2020-10-24

**Authors:** Dabok Noh, Soobin Choi

**Affiliations:** College of Nursing, Eulji University, 553 Sanseong-daero, Sujeong-gu, Seongnam-si, Gyeonggi-do, 13135, Korea; soobin.choi@mail.utoronto.ca

**Keywords:** adolescents, family conflict, homeless, intervention mapping, mental health, runaway

## Abstract

The mental health and related quality of life of runaway adolescents are global public health issues. As few intervention studies have considered the family contexts of runaway adolescents, we aimed to develop an intervention tailored specifically to the needs of this population using an Intervention Mapping protocol. First, a literature review and interviews with runaway adolescents and youth shelter workers were conducted to create a logic model of the problem. Second, the behavioral and environmental outcomes were set to adapt to stressful situations and enable families to become more resourceful in dealing with family adversity, based on the results of needs assessment. Performance objectives and changeable determinants were also created by reviewing the pertinent theories and studies. Third, theory- and evidence-based methods to influence changes in the determinants were identified. Fourth, we designed an eight-session family-based mental health program incorporating individual and family approaches for runaway adolescents. Fifth, we determined that mental health nurses at community mental health centers linked to youth shelters would serve as the program implementers. Finally, we planned a randomized controlled trial to evaluate the effects of our program on improving runaway adolescents’ mental health status and perceived family functioning.

## 1. Introduction

Runaway adolescents are at a high risk of mental health problems such as depression, post-traumatic stress disorder (PTSD), and suicidal ideation [1,2]. In addition, homeless adolescents have a higher rate of premature mortality due to suicide and substance abuse relative to the general adolescent population [3]. Previous studies indicated that runaway adolescents are not only prone to delinquent/criminal behavior but are also likely to become targets of sexual violence and prostitution [4,5].

Various factors contribute to the decision of an adolescent to run away from their home. In many cases, adolescents wish to escape an unsupportive, conflicted, or abusive home life or familial poverty [6,7]. In Korea, data from the Ministry of Gender Equality and Family indicate that family conflict was identified as the main motivator for running away by 70.0% of runaway adolescents [8]. In recent years, family conflict has tended to increase in frequency and complexity because of changes in family structures (e.g., divorce/separation) [9].

Family problems during childhood and adolescence are likely to lead to mental health problems. Particularly, family instability and conflict have important effects on a child’s mental health [9]. A previous study identified that family factors, including family instability and inappropriate parental attachment, can increase the morbidity associated with depression and alcohol abuse among runaway adolescents [10].

The traditional Korean family-centered culture prioritizes hierarchical order features in which authoritarian parents require children to comply with their demands. However, adolescents who have a perspective on Western individualism value individual freedom [11]. The authoritarian parenting style, which is against an adolescent’s expectations on autonomy, could lead to not only parent–adolescent conflicts [12] but also mental health problems among adolescents [13].

The capacities of psychological interventions such as art therapy [14], cognitive–behavioral therapy [15], motivational interviewing [16], and family therapy [17] to improve the mental health of runaway adolescents were investigated in previous studies. Although theoretically, these interventions were well-run programs, they were not developed systematically to reflect the real situations of runaway adolescents in Korea. Tailored and theory- and evidence-based interventions that consider the specific socio-cultural context are needed to improve the mental health of this population.

As family conflict is cited as the main reason for running away from home, programs without family engagement may be ineffective and lead to a chronic runaway status. In addition, because the levels of family functioning are correlated with mental health status among adolescents [18], family members should be included in interventions to improve the mental health of adolescents.

To address these concerns, we applied a systematic process to develop a theory- and evidence-based program with family engagement that would be tailored for runaway adolescents. This article describes the development of a family-based mental health program for runaway adolescents according to an Intervention Mapping (IM) protocol-based structured developmental process.

## 2. Materials and Methods

The IM protocol used in this study is a framework to guide the program planning process, which aids in developing theory- and evidence-based health promotion interventions, using a stepwise approach [19]. The IM protocol comprises six steps: (1) development of a logic model of the problem, (2) identification of program outcomes and objectives (logic model of change), (3) design of the program, (4) production of the program, (5) planning to implement the program, and (6) plan for evaluation [19]. The application of the IM protocol has advantages in promoting the development of tailored interventions to fit the needs of target groups and in establishing community-based interventions by allowing the participation of the key stakeholders in the program development process [20]. Several previous studies have used this protocol to develop mental health promotion programs [21,22,23].

### 2.1. Step 1. Logic Model of the Problem

In the first stage of the IM process, we identified the mental health problems and related quality of life issues faced by runaway adolescents, the behavioral and environmental factors related to these mental health and quality of life issues, and the determinants of behavioral and environmental factors. In addition, we described the context for the intervention and stated program goals. To complete these tasks, we used a comprehensive needs assessment including reviewing literature and theories, focus group interviews, and individual interviews.

To review findings from published literature for theory- and evidence-based answers to our questions, we conducted a comprehensive search of the published survey research, intervention studies, and literature reviews regarding the mental health problems and related quality of life issues experienced by runaway and homeless adolescents. In addition, we reviewed theories to understand mental health and family problems and to plan intervention.

The two focus group interviews involved convenience samples of eight shelter workers from two youth shelters providing residential shelter, food, and hygiene supplies to runaway youth. Youth shelter workers who are qualified social workers or youth counselors and who have substantial experience in counseling runaway adolescents were recruited. They discussed mental health and family relationship issues faced by runaway adolescents, the need for and feasibility of an intervention, and factors that should be considered. The approximately 1 h focus group interviews were conducted in meeting rooms at both shelters. For the individual interviews with adolescents, we used purposive sampling to ensure the inclusion of psychologically stable participants who would be able to discuss their experiences substantially. Five runaway adolescents from a youth shelter were recruited. They provided self-reported information about their mental health and family-related problems, the need for an intervention, and the type of help they want to get in an intervention. Each approximately 1 h individual interview was conducted in a counseling room at the youth shelter. All participants in the focus group and individual interviews signed a written informed consent. All interviews were audio-recorded and transcribed.

### 2.2. Step 2. Program Outcomes and Objectives-Logic Model of Change

In the second step of the protocol, we stated the expected behavioral and environmental outcomes based on the results of the needs assessment conducted in the first step. In addition, we specified performance objectives for the outcomes using theories as rationales. After selecting the important and changeable determinants of behavioral and environmental outcomes based on the results of our literature review conducted in step 1, we created a matrix of change objectives that the intervention would address.

### 2.3. Step 3. Program Design

In this third step, we selected the theory- and evidence-based change methods that we would use to influence the determinants of behavioral and environmental outcomes by reviewing the existing literature and theories of change. Subsequently, these change methods were translated into practical applications.

### 2.4. Step 4. Program Production

In the fourth step, we developed the scope and sequence of the program components, tools, and materials using information from steps one to three. We considered the program participants’ needs, feasibility, the context for the intervention including the population, setting, and community.

### 2.5. Step 5. Program Implementation Plan

In the fifth step, we aimed to prepare the adoption and implementation of the family-based mental health program by youth shelters in Korea considering the community context.

### 2.6. Step 6. Evaluation Plan

In the sixth stage, we aimed to develop an evaluation plan. Here, we specified effect evaluation questions which were corresponding with the program goal which was set based on the need’s assessment in step 1. We also determined the process evaluation indicators, the evaluation design, and methods of data collection and analyses.

## 3. Results

### 3.1. Step 1. Logic Model of the Problem

Through our literature review, we found that the mental health and related quality of life concerns of runaway adolescents comprise an important global public health problem. Compared to the general adolescent population, runaway and homeless adolescents were found to have higher rates of mental health problems such as conduct disorder, depression, anxiety, bipolar disorder, PTSD, suicidal ideation, and schizophrenia [1,6,24]. Regarding the quality of life, runaway adolescents also face a high risk of reduced emotional well-being [10], and dissatisfaction with family life [25].

Problematic alcohol use, risk behaviors [24,26], delinquency, criminal behavior, and school drop-out have been identified as behavioral factors associated with mental health problems and related quality of life issues [10,27].

Environmental factors can be classified as either interpersonal or community-level factors. At the interpersonal level, family factors such as poor communication, financial problems, poor psychological well-being of the parent or caregiver, poor emotional support at home [27], parental conflict, parental separation or divorce, parental alcohol abuse, and domestic violence [6,27] are associated with mental health problems and quality of life issues. Among runaway adolescents, relationships with peers and the presence of a caring adult are also related to mental health and quality of life issues [28]. At the community level, poor social connectedness, school and community environments, and economic opportunities can affect the mental health and quality of life of adolescents [28].

Poor self-esteem; a lack of adolescent resilience [28,29]; a lack of self-regulation, interpersonal, and/or problem-solving skills; a lack of self-efficacy [28]; poor parent–adolescent attachment [10]; a lack of family resilience; a lack of knowledge of parenting and adolescent development; parenting competency have all been identified as determinants of behavioral and environmental factors [28].

For two focus group interviews, five social workers and three youth counselors working at two youth shelters participated. They indicated that most of the runaway adolescents residing in youth shelters had experienced family-related difficulties such as parental divorce and family poverty, and were vulnerable to stress and mental health problems. They suggested the importance of providing an intervention targeted at improving the vulnerable mental health statuses and family functioning of adolescents. They also stated that an intervention should include promoting mutual understanding and emotional support between parents and adolescents, and having positive experiences with family through regular meetings. The focus groups explained that the return of adolescents to their homes may not be a possible outcome in some family cases because of financial difficulties and changes in the family structures due to parental divorce and remarriage. However, the focus groups suggested that improving the mental health status of adolescents and providing them with opportunities for positive experiences with family members are important in themselves, rather than as pathways to visible short-term outcomes such as the return of the adolescents to their homes.

Regarding the feasibility of an intervention, based on the focus group interviews, youth shelters strongly support the need for an intervention that aims to improve the mental health and family relationships among runaway adolescents. Shelters were willing to provide time and space required for the relevant intervention. In addition, the focus groups indicated that younger adolescents expressed a stronger will to reconcile with their family. However, the focus groups also had some concerns regarding encouraging adolescents’ parents to participate in an intervention owing to the parents’ lack of time and motivation attributed to their respective occupations.

The runaway adolescents who participated in individual interviews were likely to engage in risky and maladaptive behaviors in response to stress associated with family problems. They reported experiencing chronic family conflict and dissatisfaction with family life. They wanted individual mental health counseling and stated that their family members must have time to understand each other through an intervention.

Based on the results of this needs assessment, the program goal was set that mental health status and perceived family functioning are improved among runaway adolescents.

### 3.2. Step 2. Program Outcomes and Objectives—Logic Model of Change

The needs assessment in the first process resulted in the establishment of two expected outcomes for behavior and environment: Adaptation to stressful situations and family resilience as the capacity to be resourceful in dealing with family adversities.

While reviewing the relevant theories, we selected two theories providing rationales for performance objectives for the outcomes. The transactional model of stress and coping [30] was used as a basis for the behavioral outcome-related performance objectives, which were the use of effective coping strategies for stress and the seeking of help from social support networks in stressful situations. In addition, using the Walsh family resilience framework [31] as a rationale for the interpersonal environmental outcome-related performance objectives, we specified the performance objectives as the ability of family members to rebuild family relationships, communicate clearly and honestly, and collaborate to solve family problems.

Based on the results of our literature review conducted in step 1 of the IM process, attitudes, knowledge, skills, and self-efficacy were selected as determinants for the outcomes. Table 1 presents a matrix of the change objectives, which was constructed by crossing the performance objectives with the determinants and thus recording the change objectives.

### 3.3. Step 3. Program Design

We reviewed existing empirical evidence in the literature and theories of change to identify and choose theory- and evidence-based methods intended to influence changes in the determinants. As a result, we identified that motivational interviewing and cognitive reappraisal methods applied to the change objectives related to activating a positive attitude. Motivational interviewing to provide strength and motivation for positive changes [32] is used to facilitate changes in attitudes. According to the transactional model of stress and coping [30], a cognitive appraisal, which refers to the personal subjective interpretation of a situation and coping resources, influences an individual’s coping efforts and adaptation to a stressor. The ability to use cognitive reappraisal, which refers to the reframing of one’s thoughts to influence one’s responses to situations, could be a protective factor that enables an individual to adjust to stressful situations and reduce depressive symptoms [33]. To enhance knowledge, we derived the method of consciousness-raising from the transtheoretical model, which refers to increasing awareness about a specific problem behavior [34]. Skills training and guided practice methods were selected to enhance skills. Social modeling, emotional state improvement, and verbal persuasion were derived from Bandura’s theory [35] and selected to enhance self-efficacy. Table 2 lists the theory- and evidence-based methods and practical applications.

### 3.4. Step 4. Program Production

By integrating information from steps one to three, we developed a family-based mental health intervention for runaway adolescents aged 12–18 years who reside in youth shelters. Based on the results of needs assessment in step 1, we integrated the individual approach and family approach in the intervention. Considering the vulnerability of mental health among runaway adolescents, we consisted that adolescent individual sessions would precede family sessions. The focus group interviews conducted in step 1 revealed that most runaway adolescents residing in shelters experienced poverty, and adolescents’ parents lack time and motivation to participate in an intervention as they work to address financial difficulties. Therefore, considering the feasibility of the expected degree of family participation, an intervention format comprising four individual sessions with the adolescent and four subsequent family sessions in which the adolescents and their family members can engage together was considered appropriate. Hence, an eight-session program will be delivered in a shelter-based setting during a two-month period, and each session is expected to require approximately 60–90 min. Although the program was structured, the number of actual program sessions can be increased or decreased to accommodate the needs of adolescents and their families.

Based on the program plan from steps 2 and 3, the program components, tools, and materials were designed to ensure the achievement of the change objectives and effective operationalization of the methods and practical applications. In the first session, program engagement and motivational establishment through motivational interviewing techniques are the major emphases. In the second session, the cognitive reappraisal method is used to change the negative attitudes or beliefs influencing the adolescent’s negative emotions or behaviors on stressful events into positive attitudes or beliefs. In the third session, the participants are trained to cope effectively with stressful situations. The fourth session includes practical applications such as the provision of information about helpful resources, role-playing, mentor–mentee activities, and strong encouragement and support to encourage adolescents to seek help from social support networks in stressful situations.

The fifth to eighth sessions of the intervention will involve the family. The fifth session aims to improve family engagement and motivation for change. The sixth session includes the positive reframing of family adversities, education regarding differences between adolescents’ developmental needs and parental needs, and expressions of empathy and support to reconcile and rebuild the wounded family relationships. In the seventh session, family members are trained to communicate clearly and honestly. In the eighth session, family members identify family strengths, acquire problem-solving and decision-making skills, and practice these skills through family meetings to acquire family competence in collaborative problem-solving. Detailed information about the program components is presented in Table 3.

### 3.5. Step 5. Program Implementation Plan

We considered the community context to develop an implementation plan. Youth shelters refer runaway adolescents who need help with their mental health to community mental health centers, and adolescents residing in youth shelters are the subjects of the services provided by these centers. Therefore, to enable adoption, implementation, and maintenance of the developed program, the program implementers will comprise psychiatric and mental health nurses working at community mental health centers which were associated with youth shelters.

The psychiatric and mental health nurses at the community mental health centers will visit the youth shelters and deliver the program in counseling rooms. They will approach adolescents with cooperation from social workers at shelters and provide a program tailored to the characteristics of each adolescent.

The program implementers will receive education, training, and supervision by a nursing professor and principal investigator, and will provide the program to adolescents according to the program protocol. To maintain the quality of the services, the nursing professor and principal investigator will hold regular once-weekly meetings with the program implementers and youth shelter workers. Partnerships will be established between youth shelter workers and nurses from community mental health centers to enable cooperation.

### 3.6. Step 6. Evaluation Plan

According to the program goal, which was set based on the need’s assessment in step 1, the hypotheses will test whether the mental health status and perceived family functioning improved in the intervention group relative to the comparison group. The primary outcome will be the effect of the program on mental health issues including depression, internalizing and externalizing behaviors, psychological distress, and suicidality among adolescents. These indicators were determined based on a review regarding outcome variables used in previous experimental studies of runaway and homeless adolescents. The secondary outcome will be the effect of the program on perceived family functioning among adolescents. The process evaluation should address the reach (percentage of the intended participants who used the program) and the dose of the intervention (average dose received by program participants). We will evaluate whether the needs of participants, as assessed in step 1, will be satisfied, and the new information and perspective generated in the evaluation process of the program will help us revise the previous steps in the IM process.

The effectiveness of the developed program will be evaluated in a randomized controlled trial (RCT). The participants will be divided into experimental and comparison groups via computer-generated random allocation. The participants that meet the following criteria will be included: runaway adolescents residing in youth shelters and 12–18 years of age. The exclusion criteria will be as follows: (1) adolescents diagnosed with an intellectual disability or specific learning disorder that can impair their ability to understand the intervention procedure and (2) those who are currently receiving other psychiatric treatments. In addition, family members currently receiving treatment for acute psychiatric symptoms will not be allowed to participate in the family sessions.

The participants’ data will be collected using self-administered questionnaire surveys, which will be completed individually in quiet counseling rooms to protect the participants’ confidentiality. The assessment time points will include the baseline (before the program) and immediately and 1 month after program completion.

The data will be analyzed statistically using IBM SPSS Statistics for Windows, version 26.0 (IBM Corp., Armonk, NY, USA), and the intervention effects will be examined using a one-way repeated measure multivariate analysis of variance (MANOVA). A power analysis conducted using the G * Power program [36] indicated that a total sample of 211 subjects would be needed to detect a medium effect (*f* = 0.25) with 80% power using MANOVA at an alpha level of 0.05. Therefore, we will collect data from 118 participants each in the experimental and comparison groups (*N* = 236) to accommodate the expected attrition of 10% over the three-month period from the baseline assessment to the final evaluation.

## 4. Discussion

In this article, we have described the systematic development process of a family-based mental health program for runaway adolescents residing in youth shelters. Although previous studies conducted theory- and evidence-based psychological interventions for this population [15,17], to our knowledge, only a few studies conducted tailored interventions that considered their family context among runaway adolescents in Korea. The strengths of the IM protocol used in this study include the application of a structured and systematic development process, the development of a tailored program that considers the socio-cultural context and stakeholders, and an emphasis on theory- and evidence-based intervention [19]. Therefore, we used the IM protocol to develop a tailored theory- and evidence-based program for runaway adolescents in Korea, despite it being a time- and effort-consuming process.

The first strength of our developed program involves its basis on a comprehensive needs assessment, which combined a literature review and the results of interviews with runaway adolescents and youth shelter workers. From an ecological perspective, the needs assessment identified the individual and family factors influencing the mental health problems of runaway adolescents. We also identified the youth shelters where runaway adolescents reside as appropriate settings in which to implement the intervention. These findings led to the design of our program to incorporate individual sessions and family sessions within a shelter setting.

The second strength of our developed program is its emphasis on a theoretical framework in combination with the need’s assessment. In this study, the transactional model of stress and coping [30] and the Walsh family resilience framework [31] provided the rationales for the behavioral outcome- and interpersonal environmental outcome-related performance objectives. We also used theory- and evidence-informed decisions to select effective change methods. We expect that this approach will help to close the gaps among the scientific evidence, practice, and policy in the field of community mental health.

Our study differs from prior studies because we identified psychiatric and mental health nurses at community mental health centers linked to youth shelters as the program implementers. A systematic review study identified that the program implementers in previous intervention studies of homeless and runaway adolescents were research investigators, such as counselors, nurses, psychologists, and clinicians [37]. When considering how to enable the adoption, implementation, and maintenance of the program, however, we considered that youth shelters refer adolescents with mental health issues to community mental health centers, where the cases are managed by the nurses. By considering the system linking youth shelters and community mental health centers, this program implementation plan will help to maintain the program itself, as well as the initial adoption and implementation.

The IM protocol provided a useful framework in developing a program to target runaway adolescents in this study, suggesting the application of the IM protocol in the field of mental health promotion. For this program, we adopted an individual approach, which enabled customization to the individual needs and circumstances of runaway adolescents, and a family intervention modality, which was intended to intervene in family problems because these were identified as the main cause underlying runaway behavior. The effectiveness of the developed program on mental health status and perceived family functioning of runaway adolescents remains to be demonstrated in further RCTs.

### Limitations

This study had several limitations. First, although we used a comprehensive needs assessment including reviewing literature and theories, focus group interviews, and individual interviews, limited numbers of participants were interviewed because of recruiting only participants from the participating shelters involving in our development process. Second, although this study implemented the IM protocol to consider environmental and individual factors via an ecological approach, the developed program did not combine environmental components beyond the family level. Other environmental factors such as school and society factors may have important effects on the mental health and quality of life of runaway adolescents. Therefore, further studies with a more comprehensive approach that incorporates school and societal efforts are needed. Third, this study set the overall change objectives in consideration of the runaway and family contexts of the target population. However, it may be necessary to set detailed change objectives according to the mental health status and family conflict level experienced by each adolescent.

## 5. Conclusions

The IM protocol-based program development in this study yielded a theory- and evidence-based intervention that was tailored specifically to the needs of runaway adolescents residing in youth shelters. The program incorporated both individual and family sessions developed in consideration of the runaway and family contexts. In addition, this program will be provided by psychiatric and mental health nurses at community mental health centers linked to youth shelters. In the future, an RCT will be conducted to evaluate the effects of this program based on our evaluation plan.

## Figures and Tables

**Table 1:** Matrix of behavioral and environmental outcomes.

Outcomes	Performance Objectives	Determinants			
		Attitudes	Knowledge	Skills	Self-Efficacy
Behavioral Outcome: Adolescents Adapt to Stressful Situations	PO.1. Adolescents use effective coping strategies for stress.	A.1. Adolescents express a more positive perspective of stressful situations.	K.1. Adolescents list effective coping strategies.	SK.1. Adolescents demonstrate and apply effective coping strategies.	SE.1. Adolescents express confidence in using effective coping strategies.
	PO.2. Adolescents seek help from social support networks in stressful situations.	A.2. Adolescents express positive attitudes toward seeking help.	K.2.a. Adolescents list available helping resources. K.2.b. Adolescents identify benefits of seeking help from formal resources.	SK.2. Adolescents demonstrate help-seeking behavior in stressful and risky situations.	SE.2. Adolescents express confidence in help-seeking behavior.
Environmental outcome: Families Become More Resourceful in Dealing with Family Adversities	PO.3. Family members rebuild family relationships.	A.3.a. Family members recognize meanings of family adversities. A.3.b. Family members express positive attitudes toward reconciliation of wounded relationships.	K.3.a. Family members identify individual needs and differences K.3.b. Family members identify ways to express mutual empathy and support.	SK.3. Family members demonstrate mutual empathy and support.	SE.3. Family members express confidence in reconciliation of wounded relationships through mutual empathy and support.
	PO.4. Family members communicate clearly and honestly.	A.4. Family members express positive feelings about family communication.	K.4. Family members identify ways to communicate clearly and honestly with other family members.	SK.4. Family members demonstrate ability to communicate clearly and honestly.	SE.4. Family members express confidence in ability to communicate clearly and honestly.
	PO.5. Family members collaborate to solve family problems.	A.5.a. Family members recognize family strength. A.5.b. Family members express proactive stance for collaborative problem solving and decision making.	K.5. Family members identify ways of collaborative problem solving and decision making.	SK.5. Family members demonstrate collaborative problem-solving and decision-making skills.	SE.5. Family members express confidence in collaborative problem solving and decision making.

**Table 2:** Changeable determinants, theory- and evidence-based change methods, and practical applications.

Determinants	Methods	Practical Applications
Attitude	Motivational interviewing	Strengthen personal motivation for and commitment to positive changes
	Cognitive reappraisal	Use cognitive reappraisal to reframe one’s thoughts in a more positive perspective
Knowledge	Consciousness-raising	Provide information and feedback
Skills	Skills training	Develop skills from activity-based learning
	Guided practice	Role-play in multiple scenarios
Self-efficacy	Social modeling	Mentor–mentee activities
	Improving emotional states	Relaxation training
	Verbal persuasion	Provide strong encouragement

**Table 3:** Scope and sequence of a family-based mental health program for adolescents residing in youth shelters.

Intervention Modality	Session	Contents	Methods, Practical Applications, Activities
Individual Adolescent Session	1	Engagement and establishing motivation for program	Establishing the therapeutic alliance Establishing motivation for change
	2	Positive thinking: A (Antecedent events), B (Beliefs or thoughts), and C (Consequences)	Identifying stressful events, automatic thoughts, and responses (emotions or behaviors) Modifying negative thoughts to more positive thoughts Practice positive self-talk
	3	Coping with stressful situations	Providing information and feedback about coping strategies Coping skills training Relaxation training
	4	Asking for help to handle stressful situations	Providing information about stressful and risky situations and helping resources Role-play in multiple scenarios Mentor–mentee activities Providing strong encouragement and support
Family Session	5	Family engagement and establishing motivation for change	Family engagement activities Establishing the therapeutic alliance Establishing motivation for change
	6	Rebuilding relationships with family	Positive reframing of family adversities Providing information and feedback about adolescents’ developmental needs and parental needs Empathy skills training Practice interacting positively with each other
	7	Improving family communication	Communication skills training Family role-play Providing strong encouragement
	8	Improving collaborative problem solving	Highlight family strengths Skills training in collaborative problem solving and decision making Family meetings
